# Restrictive measure for the commercialization of antimicrobials in Brazil: results achieved

**DOI:** 10.11606/s1518-8787.2019053000879

**Published:** 2019-08-15

**Authors:** Josiane Moreira da Costa, Cristiano Soares de Moura, Cristiane Aparecida Menezes de Pádua, Aline Siqueira Fogal Vegi, Sérgia Maria Starling Magalhães, Marina Barra Rodrigues, Andréia Queiroz Ribeiro

**Affiliations:** I Universidade Federal de Minas Gerais. Faculdade de Farmácia. Programa de Pós-Graduação em Medicamentos e Assistência Farmacêutica. Belo Horizonte, MG, Brasil; II McGill University. Division of Clinical Epidemiology. Department of Epidemiology. Montreal, Quebec, Canada; III Universidade Federal de Minas Gerais. Faculdade de Farmácia. Departamento de Farmácia Social. Belo Horizonte, MG, Brasil; IV Universidade Federal de Viçosa. Centro de Ciências Biológicas e da Saúde. Departamento de Nutrição e Saúde. Viçosa, MG, Brasil

**Keywords:** Anti-Bacterial Agents, Prescription Drug Overuse, legislation & jurisprudence, Drug Resistance, Microbial, Drug Monitoring, Pharmacovigilance

## Abstract

**OBJECTIVE:**

To assess whether the incidence of hospital infection by a resistant microorganism decreased after the implementation of the restrictive measure of the National Health Surveillance Agency for the commercialization of antimicrobials.

**METHODS:**

A historical cohort study of medical records of adult patients admitted to a general and public hospital from May 2010 to July 2011. A cohort was formed with patients admitted in the period before the restrictive measure for the commercialization of antimicrobials (Phase I) and a second cohort was formed with patients admitted after the implementation of the restrictive measure (Phase II).

**RESULTS:**

The instantaneous risk of hospital infection by a resistant microorganism was estimated at seven by 1,000 people-time (95%CI 0.006–0.008) in Phase I, and four by 1,000 people-time (95%CI 0.003–0.005) in Phase II of the study. The differences between the survival curves in the different phases of the study and stratified by age group were also significant (p < 0.05).

**CONCLUSIONS:**

The results suggest that the implementation of the restrictive measure of the commercialization of antimicrobials by the National Health Surveillance Agency reduced the incidence of hospital infection by a resistant microorganism.

## INTRODUCTION

Bacterial resistance to antimicrobials (ATM) poses a serious threat to global public health. Despite its multifactorial genesis, one of the main factors that triggers it is the unnecessary and abusive use of ATM, a fact extensively described in the international and national literature^1^.

Resistance can decrease the effectiveness of ATM, requiring the administration of second line drugs, which are usually more toxic and costly, causing damage to the patient and increasing the hospitalization time and expenses for the individual and for the health system^5,6^.

Specific characteristics of developing countries favor the occurrence of microbial resistance in these places, such as low hygiene conditions, poor living conditions, poor quality of medications and access to the parallel market of ATM^7^. An increase in the consumption of ATM in emerging countries is identified, with Brazil being among the five countries with the highest indices between 2000 and 2010^8^. Studies on microbial resistance published in the country so far present incipient data^9^, but show expressive increase in the resistance of these organisms and, consequently, increased morbidity and mortality and the cost of infections^10,11^.

Microbial resistance can be developed by selective pressure resulting from exposure to ATM and from the exchange of genetic material among several species of microorganisms. In this context, exposure to ATM is a fundamental factor in the selection of resistant species. Among the strategies to minimize the development of resistance, the reduction of the prescription of ATM and the implementation of strategies that stimulate rational use at Community level or in hospitals have deserved prominence^5,6,12^. The optimization^a^ of the use of ATM is among the five objectives of the Global Action Plan of the World Health Organization (WHO) to control microbial resistance^13^, in order to reduce the supply and overprescription of antibiotics for human and veterinary use, as well as to strengthen the regulation of its use worldwide. In Brazil, up to 2010 the ATM could be acquired only with the presentation of the prescription, limiting the supervision and favoring self-medication. The collegiate board of the National Health Surveillance Agency (ANVISA), through RDC 44, published on October 26, 2010, in its Art. 2nd determined that prescribed antibiotics can only be dispensed with special control prescription, aiming to restrict the free access to these drugs and minimize the occurrence of microbial resistance^14^.

Inadequate use of ATM at Community level contributes to the selection of resistant strains and dissemination of resistance genes^15^. Since studies indicate that its use in hospitals affects the community microbial resistance and vice versa^4,16,17^, it is relevant to evaluate the impact of the implementation of restrictive measures for the marketing of these medicines in both environments.

In the literature, scientific publications indicate the influence of the restrictive measure in the consumption of ATM in Brazil^18,19^, but no publications were identified on the impact of this measure on the reduction of microbial resistance in the hospital environment. Given this context that hospital infections raise health expenditures and cause harm to patients, our study aimed to compare the occurrence of microbial resistance in a hospital environment before and after the implementation of the restrictive measure of ANVISA for the commercialization of ATM in Brazil.

## METHODS

### Study Design, Location and Population

This is a historical cohort study including adult patients admitted to the Hospital Risoleta Tolentino Neves (HRTN) in the period from May 2010 to July 2011. HRTN is a general public emergency hospital that has about 330 beds, being a reference for the northern region of Belo Horizonte and neighboring municipalities. Approximately 10,000 patients are attended each month, most are attended in the emergency room and about 13.0% of the patients are hospitalized in the same institution.

We included patients over 18 years of age, for whom culture exams were requested due to suspected hospital infection or routine procedures to identify bacterial colonization. Patients with diagnosis of bacterial infection at admission or up to 72 hours after hospitalization, women hospitalized for childbirth and puerperium, and patients transferred from another hospital or with length of stay of less than 72 hours were excluded.

This study consisted of two cohorts: The first included patients admitted to the HTRN from May to October 2010, period before the restrictive measure for the commercialization of ATM (Phase I), and the second was formed by patients admitted between February and July 2011, after the implementation of the measure of ANVISA (Phase II). The option for this period aimed to obtain symmetrical time intervals that minimized the interference of non-controllable factors such as turnover of professionals, shortage of the supply of medications and influence of non-standardization of procedures to conduct the examinations of the institution. The team responsible for controlling infections associated with health care at the institution and the clinical body did not undergo alterations during the study period, but a change was recorded in the laboratory that performed the exams in the HRTN in the second semester of 2011 (period after data collection). No shortage of supply of medications occurred during the study periods.

### Definition of Study Variables

The outcome of interest was infection or hospital colonization by a microorganism resistant to ATM, evidenced by positive results of *in vitro* culture of microorganisms and result of the sensitivity test to Antimicrobial agents (*in vitro* antibiogram), interpreted as “resistant” in patients with hospitalization stay exceeding 72 hours^20^. In this study, microbial resistance was defined as the antibiotic resistance from a clinical point of view, considering a higher probability of therapeutic failure when an infection by a given microorganism is treated with a class of antibiotics customarily used in clinical practice^21^, identified by resistance results in the antibiogram.

The predictor variables were the phases of the study (before and after the implementation of the restrictive measure), age (adults < 60 years old and older adults ≥ 60 years), sex and risk profile of the patients. In relation to the last variable, the hospital under study has a management process in which patients are classified according to the following clinical risk profiles: critically ill patients (assisted in the polytrauma care sector and in the intensive care center), patients in the medical clinic (usually hospitalized for worsening chronic health problems), surgical patients (requiring surgical intervention), and maternal-infant profile (maternity and pediatrics). As the patients treated in the maternity and pediatrics sectors did not present inclusion criteria, the following risk profiles were considered in the study: critically ill, in the medical clinic and surgical patients. The clinical profile was considered a proxy variable of clinical severity or of performing invasive procedures of greater impact.

We also identified the total number of culture exams requested by the patient, the resistant microorganisms and the resistance profile. The microorganisms were classified as producers of extended spectrum β-lactamases (ESBL) when the isolate was a producer of extended spectrum β-lactamase. Those classified as *Klebsiella pneumoniae carbapenemase* (KPC) were those whose strains were carbapenemase-producing, identified by the positive Hodge Test^22^. Also according to the recommendations of the institutional protocol, the growth of atypical microorganisms and fungi in blood culture was counted as resistance when considering the severity of the clinical consequences of these situations. In case of the result “microbial growth in mixed culture,” the examination was repeated.

The procedures of culture collection in the HRTN occurred in case of clinical suspicion of infection or through the culture of axillary and anophanous swab, whose institutional protocol recommended weekly collection for patients with hospitalization time exceeding 15 days, even without signs or symptoms of infection.

### Data Collection and Analysis

The information on bacterial resistance and predictor variables was collected from secondary data, through the review of the patient’s electronic record and the generation of computerized reports.

Regarding the identification of microbial resistance, a computerized report of all the culture exams performed for the patients under study was generated, then the results were checked. In specific cases, the information was complemented with records of the hospital infection control committee on the resistance profile in the institution.

Descriptive analysis of the variables was performed for the study population, with distribution of absolute and relative frequencies and measures of central tendency and variability. Survival curves estimated by the Kaplan-Meier method were used to compare the time until the occurrence of at least one hospital infection record by a resistant microorganism in the two phases of the study. The same method was used to compare differences between the study phases stratified by age group. The free time of the outcome was calculated between the date of hospital admission and the occurrence of the first episode of the outcome of interest, censored by the occurrence of death or by hospital discharge in both phases of the study. The Cox proportional hazards model was used to estimate the incidence density ratio (*hazard ratio*) for microbial resistance infection (MRI) according to the predictor variables. For all analyses, a 0.05% significance level was adopted. All analyses were conducted using the Stata statistical software version 13.0.

### Ethical Considerations

The project was approved by the Human Research Ethics Committee of the Federal University of Viçosa (Opinion 176/2012).

## RESULTS

During the study period, 5,178 hospitalizations for 4,786 patients in Phase I and 4,618 hospitalizations for 4,261 patients in Phase II were analyzed. Approximately 53.0% of the patients were men (52.1% and 53.0% in Phases I and II, respectively), with a mean age of 49.2 (SD = 20.8) years in Phase I and 49.7 (SD = 21.2) years in Phase II (p > 0.05 for sex and age).

From the total of patients monitored in Phase I, culture exams were requested for 922 patients (19.3%), and 8,149 exams were performed, with an average of 8.8 exams per patient. In Phase II, exams were requested for 684 (14.8%) patients, and a total of 3,404 exams were performed, corresponding to an average of 5.0 exams per patient.

Of the total tests performed, 1,803 and 1,130 isolates were identified in Phases I and II, respectively. Approximately 1,109 (62%) isolates in Phase I showed resistance, while in Phase II, resistance was identified in 381 (34%) isolates. In Table 1, resistant microorganisms are described by phase.

**Table 1:** Resistance profile of microorganisms by study phase.

Resistant microorganism	Phase I (n)	Phase II (n)	Resistance profile
*Acinetobacter baumannii*	308	124	Imipenem or Meropenem or Ceftazidime or Ceftriaxone or Cefotaxime or Cefepime
*Achromobacter* sp.	1	0	Imipenem or Meropenem or Ceftazidime or Ceftriaxone or Cefotaxime or Cefepime
*Alcaligenes faecalis*	2	0	ATYPICAL*
*Candida* sp.*	8	9	ATYPICAL*
*Citrobacter* sp.	1	4	Imipenem or Meropenem or Ceftazidime or Ceftriaxone or Cefotaxime or Cefepime
Carbapenemase-producing *Enterobacter aerogenes*	1	0	Meropenem and imipenem
*Enterobacter* sp.	54	16	Imipenem or Meropenem or Ceftazidime or Ceftriaxone or Cefotaxime or Cefepime
*Enterococcus* sp.	138	21	Vancomycin
*Escherichia coli*	20	25	Ceftazidime or Ceftriaxone or Cefotaxime or Ciprofloxacin or Levofloxacin or Gatifloxacin or Cefepime
*ESBL*-producing *Escherichia coli*	11	1	Beta-lactams
*Geotrichum candidum*	1	0	ATYPICAL*
*Haemophilus* sp.	1	0	ATYPICAL*
*Klebsiella* sp.	35	12	Imipenem or Meropenem or Ceftazidime or Ceftriaxone or Cefotaxime or Cefepime
ESBL-producing *Klebsiella pneumoniae*	19	0	Beta-lactams
Carbapenemase-producing *Klebsiella pneumoniae*	5	2	Beta-lactams
*Morganella morganii*	5	4	Imipenem or Meropenem or Ceftazidime or Ceftriaxone or Cefotaxime or Cefepime
*Proteus mirabilis*	23	53	Imipenem or Meropenem or Ceftazidime or Ceftriaxone or Cefotaxime or Cefepime
ESBL-producin *Proteus mirabilis*	0	3	Beta-lactams
*Providencia stuartii*	2	3	Imipenem or Meropenem or Ceftazidime or Ceftriaxone or Cefotaxime or Cefepime
*Pseudomonas aeruginosa*	133	46	Ciprofloxacin or Levofloxacin or Imipenem or Meropenem or Ceftazidime or Piperacillin or Cefepime
*Salmonella* group	1	0	ATYPICAL*
*Serratia* sp.	11	3	Imipenem or Meropenem or Ceftazidime or Ceftriaxone or Cefotaxime or Cefepime
Sphingomonas paucimobilis*	2	0	Sulfamethoxazole + Trimethoprim
*Staphylococcus aureus*	228	10	Oxacillin
*Staphylococcus* sp.	21	17	Oxacillin
*Staphylococcus epidermidis*	33	19	Oxacillin
*Staphylococcus haemolyticus*	23	6	Oxacillin
*Coagulase-negative staphylococcus*	8	3	Oxacillin
Stenotrophomonas maltophilia*	5	0	Sulfamethoxazole + Trimethoprim
*Streptococcus agalactiae* (beta-hemolytic) of group B	3	0	Penicillin or Ceftazidime or Ceftriaxone or Cefotaxime or Cefepime
*Streptococcus pneumoniae*	1	0	Penicillin or Ceftazidime or Ceftriaxone or Cefotaxime or Cefepime
*Streptococcus pyogenes*	2	0	Penicillin or Ceftazidime or Ceftriaxone or Cefotaxime or Cefepime
Non-pneumococcus *Streptococcus* sp. (alpha-hemolytic)	3	0	Penicillin or Ceftazidime or Ceftriaxone or Cefotaxime or Cefepime
Total resistants	1,109	381	

ESBL: Extended spectrum beta-lactamase

* Microorganisms considered atypical because they are not incorporated into the hospital microbiota. According to the institutional protocol, they are counted as resistant, considering the severity of the clinical consequences of infections and the need to implement surveillance strategies to prevent future infections by these pathogens. As to fungi, they were counted as resistant in cases of blood infections.

**Figure 1:**
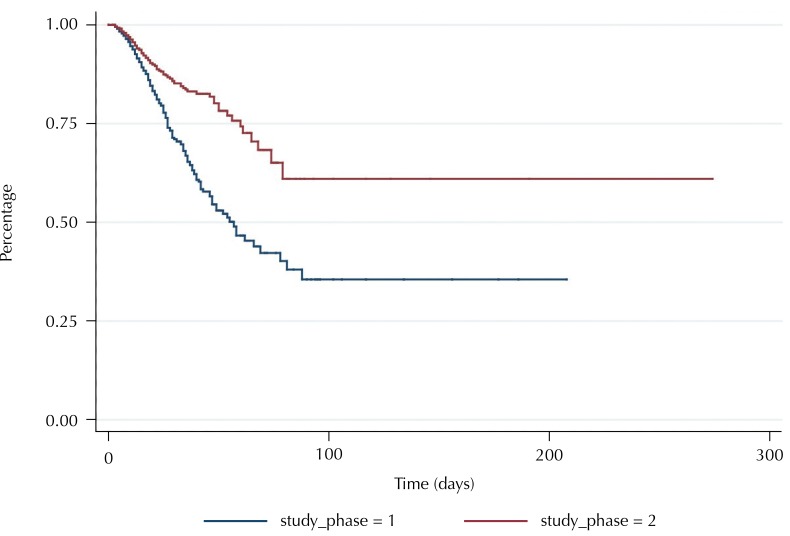
Survival curve free from microbial resistance for hospitalized patients. Belo Horizonte, MG, 2010–2011.

The incidence density of hospital infection by a resistant microorganism (in number of cases per hospitalization/day) was significantly higher in Phase I (7 for 1,000 people-time, 95%CI 0.006–0.008) compared with Phase II (4 for 1,000 people-time, 95%CI 0.003–0.005). We observed that the free time of infection by bacterial resistance of 75% of the study population was 27 days in Phase I and 60 days in Phase II (Figure 1).

**Figure 2:**
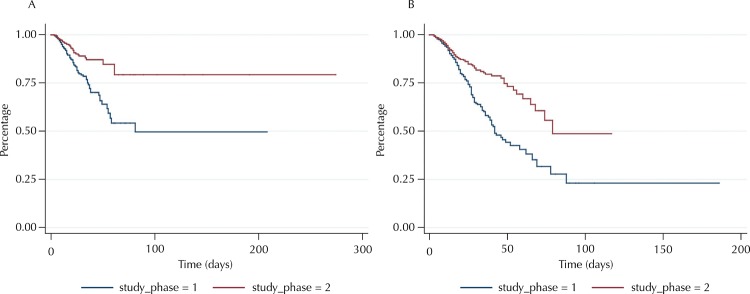
Survival curve free from microbial resistance for (A) adult patients and (B) hospitalized older adults. Belo Horizonte, MG, 2010–2011.

**Figure 3:**
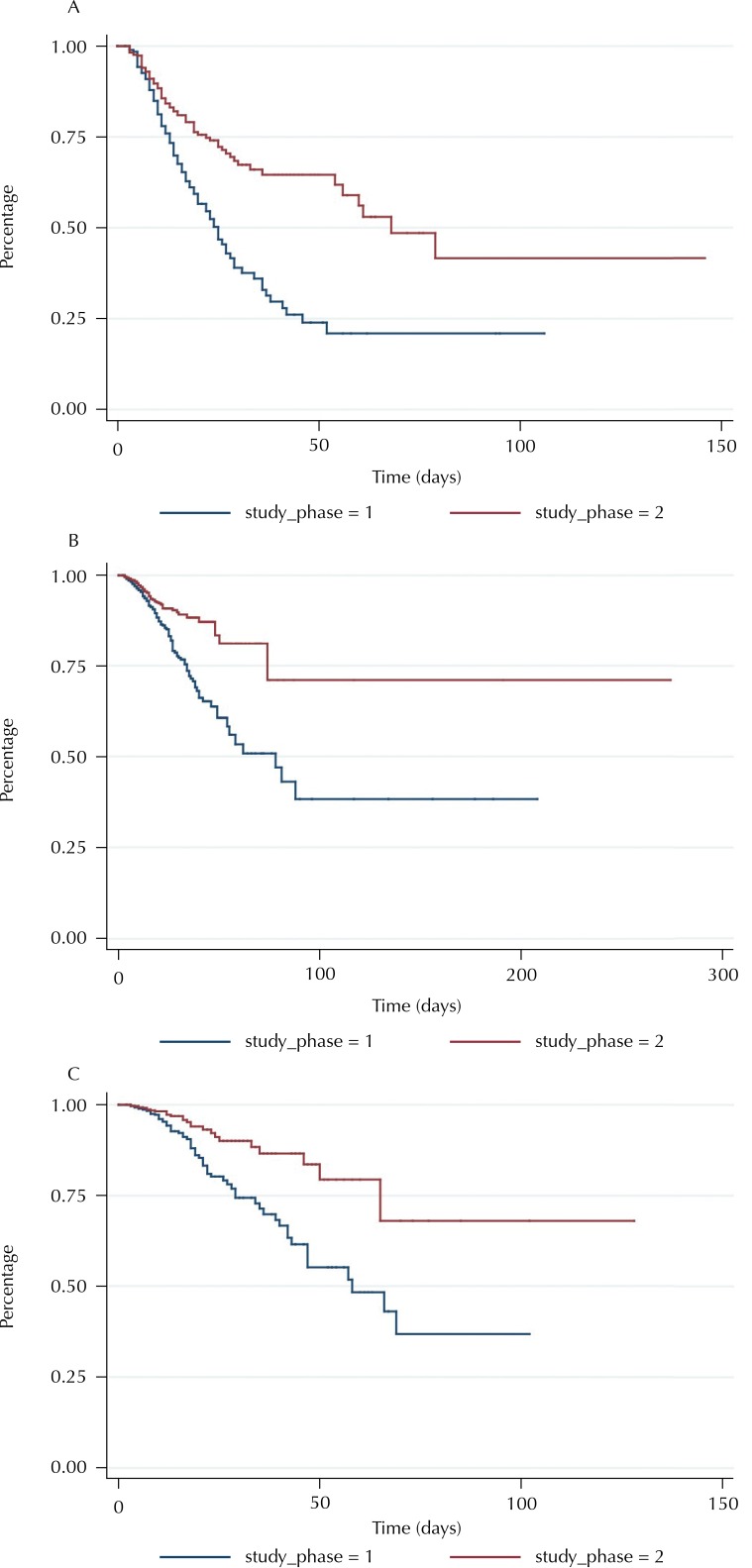
Survival curve free from microbial resistance for (A) critically ill, (B) in the medical clinic and (C) surgical patients. Belo Horizonte, MG, 2010–2011.

In the stratified analysis by age group, differences were found between the instantaneous risk in the study phases for both groups (Log-rank, p < 0.05). In adults, the instantaneous risk was five for 1,000 people-time (95%CI 0.004–0.006) in Phase I and three for 1,000 people-time (95%CI 0.002–0.004) in Phase II. In older adults (60 years or more), in Phase I it was 0.010 (95%CI 0.008–0.011) and in Phase II it was 0.006 (95%CI 0.005–0.007). The free time of MRI in 75% of the adults was 36 days in Phase I, while in Phase II the outcome was observed in less than 25% of them. The free time of MRI in 75% of the older adults was 25 and 48 days in Phases I and II, respectively. In both age groups, the differences in the free time of MRI between the phases were statistically significant (Figure 2). The free time of MRI was lower in Phase I compared with Phase II for those in the medical clinic, surgical and critically ill profiles (Log-rank, p < 0.05) (Figure 3).

In the multivariate analysis, we found that age, study phase and risk profile remained independently associated with the risk of MRI. Controlling by age and risk profile, the risk of MRI in Phase II was approximately 50% lower than the same risk in Phase I (Table 2).

**Table 2:** Final model of the association between study phase, age, risk profile and MRI. Belo Horizonte, MG, 2010–2011.

Variable	HR (95%CI)	

	Unadjusted (95%CI)	Adjusted*
Study phase		
I	1.0	1.0
II	0.54 (0.45–0.65)	0.46 (0.39–0.56)
Age group (years)		
Adult (< 60)	1.0	1.0
Older adult (≥ 60)	1.64 (1.37–1.97)	1.56 (1.30–1.88)
Risk profile		
Critically ill patients	1.0	1.0
Medical clinic	0.33 (0.27–0.40)	0.29 (0.23–0.35)
Surgical clinic	0.36 (0.28–0.46)	0.34 (0.27–0.44)

HR: hazard ratio; MRI: microbial resistance infection

* adjusted for study phase and age group.

## DISCUSSION

This study shows the contributions of the restrictive measure in reducing the incidence density of nosocomial infections in a population of older patients in a general hospital in Brazil, even after adjusting for age. These findings are important to the scientific literature, considering that, although this measure was adopted in 2010, little is known about its impact on microbial resistance in the hospital environment.

Few Brazilian studies have been conducted in order to identify the contributions of the restrictive measure in the control of infections, and most of them are directed to the analysis of the sales of ATM in drugstores and decrease in the resistance in the community environment^18,19^. We understand that one of the intuitiveness of the measure was also the decrease in the occurrence of microbial resistance in the hospital environment and we believe that the results of this study have an innovative character.

This study showed that the restriction to the community use of ATM can reduce hospital infections by resistant microorganisms. In a hospital environment, microbial resistance is aggravated by different factors, such as fragility of patients’ health conditions and proximity to beds, which facilitates the dissemination of cross-Infection^5^. To understand the interference of the community use of ATM in this process favors the implementation and improvement of preventive actions, such as the determination of control over the sale of ATM normalized by ANVISA through RDC 44/2010^14^.

The results obtained are not restricted to a single hospitalization unit, but comprise different clinics of a large teaching hospital, representing a diversified spectrum of health conditions that differentiate patients from the susceptibility to MRI.

The criterion used to define infection as of hospital nature was its registration in a period equal to or greater than 72 hours of hospital stay, in order to exclude cases of community infection. This criterion is defined in Brazil by Ordinance 2,616 of May 12, 1998^21^ and has been adopted in different studies^23,24^. It is not possible to guarantee that some cases detected have not been originated in the community.

According to our results, the risk of MRI was lower in the post-implementation phase of the restrictive measure, even after adjusting for age. This scenario suggests a reduction or greater adequacy of the prescription of ATM in the community, which may have been reflected in the cited hospital in the second phase of the study. However, studies indicate that, although the implementation of strategies to reduce the consumption of ATM is beneficial and can reduce bacterial resistance^16^, the decrease in consumption may not mean a decrease in infections by multidrug-resistant microorganisms, which should also involve the rational prescription and the decrease in the consumption of specific classes of ATM^16,17^. Additionally, changes in the behavior of health professionals regarding preventive measures of hospital infection may have occurred simultaneously with the reduction in the use of ATM, contributing to the reduction in MRI. In the analyzed period, no records or reports of restructuring or drastic changes were identified in the routines of the hospital control and infection commission in a study that justified the reduction in the internal consumption of ATM. Presuming that this assumption is valid, the influence of the immediate impact of ANVISA’s resolution on the rationalization of the use of ATM and the adoption of measures to prevent hospital infection in Phase II should be considered.

Several studies indicate the abusive use, without indication and in inadequate doses as potentiator of the emergence of strains of microorganisms resistant to ATM^5,25,26^. However, it is noteworthy that the actions to control and decrease infections by resistant microorganisms are complex and should contemplate not only the restriction of the sale of ATM through medical prescription, but other strategies not addressed in RDC 44/2010, such as the implementation of educational practices for rational prescription, elaboration and implementation of protocols, supervision of prescriptions, hand sanitization campaigns, monitoring and health education to patients to guarantee rational use, control of animal and environmental use, among others^4,17,27^. These measures should be carried out not only at community level but also in the hospital. This reinforces the need for continuous assessments of drug regulatory measures, aiming at the sustainability of MRI reduction. Although the reduction in the acquisition of antimicrobials after RDC 44 was not homogeneous throughout the country, the region where the study was conducted was the one with the largest decrease^18^.

The limitations of this study include the impossibility of identifying the association between the reduction in crop collection in the analyzed times and the reduction in infections or colonizations by resistant microorganisms; the short analysis time; the impossibility of using the statistical analysis “interrupted time series,” which would be more adequate to analyze dependent samples in sequential phases in time; and the use of few control variables. Although the period to assess the impact of the restriction on the sale of antimicrobials on microorganisms of nosocomial origin may seem short, a systematic review and meta-analysis indicate the occurrence of microbial resistance between one and six months after the use of ATM^28^, which corroborates our findings.

Our study identified that the anterior and posterior phases to the restrictive measure are independently associated with the reduction of microbial resistance. This is the first investigation in Brazil suggesting the influence of restrictive measures on the reduction of this type of infection in the hospital environment. Therefore, other studies with the objective of monitoring the rates of resistance incidence and the sustainability of the restrictive measure must be conducted.
